# Extrachromosomal Circular DNA: Category, Biogenesis, Recognition, and Functions

**DOI:** 10.3389/fvets.2021.693641

**Published:** 2021-09-09

**Authors:** Xiukai Cao, Shan Wang, Ling Ge, Weibo Zhang, Jinlin Huang, Wei Sun

**Affiliations:** ^1^Joint International Research Laboratory of Agriculture and Agri-Product Safety of Ministry of Education of China, Yangzhou University, Yangzhou, China; ^2^College of Animal Science and Technology, Yangzhou University, Yangzhou, China; ^3^Jiangsu Key Laboratory of Zoonosis, Yangzhou University, Yangzhou, China

**Keywords:** eccDNA, microDNA, tumor, livestock, molecular marker

## Abstract

Extrachromosomal circular DNA (eccDNA), existing as double-stranded circular DNA, is derived and free from chromosomes. It is common in eukaryotes but has a strong heterogeneity in count, length, and origin. It has been demonstrated that eccDNA could function in telomere and rDNA maintenance, aging, drug resistance, tumorigenesis, and phenotypic variations of plants and animals. Here we review the current knowledge about eccDNA in category, biogenesis, recognition, and functions. We also provide perspectives on the potential implications of eccDNA in life science.

## Introduction

It was previously thought that genetic variation and V(D)J recombination were the main reasons for genome heterogeneity of different tissues from the same individual or different cells from the same tissue. However, recent studies have shown that extrachromosomal circular DNA (eccDNA) is an additional source of genomic heterogeneity. EccDNAs are a group of double-stranded circular DNA molecules that are derived and free from eukaryotic genome DNA. They could function in genome evolution and environmental adaptation, which depend on eccDNA sequence features. The high copy number and the significant transcriptional activity of eccDNA lead to the overexpression of the inhabiting genes (1). Additionally, eccDNAs could serve as mobile enhancers to trans-regulate chromosomal genes (2). Small eccDNAs are more widespread, but less is known about their function in cell biology. They are too small to contain protein-coding genes. MicroDNA can be released from normal and tumor tissues to plasma and serum, implying their roles in cell communication (3, 4). Transcription factor sponge is another speculated function of microDNA, where the accumulation of specific microDNA could titrate components of the replication or transcription machinery and lead to an inability to replicate or transcribe genomic DNA (5). Thus, establishing the associations of microDNA with economic phenotype or disease is an important direction for future exploration.

## History Notes of EccDNA

EccDNA was first detected and measured in pig sperm using electron microscopy technology, with a length of ~0.5–16.8 μm (1 μm ≈ 3,100 bp) (6, 7). The length of eccDNA in the Hela cell nucleus was estimated to be 0.2–19.8 μm, compared with 4.81 ± 0.24 μm (mean ± SD) of mitochondrial DNA, and the eccDNA count was 20% of the mitochondrial DNA count (8). When analyzing a set of tumor karyotypes, scientists found many small double chromatin bodies (double-minutes, DMs), sometimes in large numbers, in addition to the apparently structurally intact chromosome (9).

**Figure 1:**
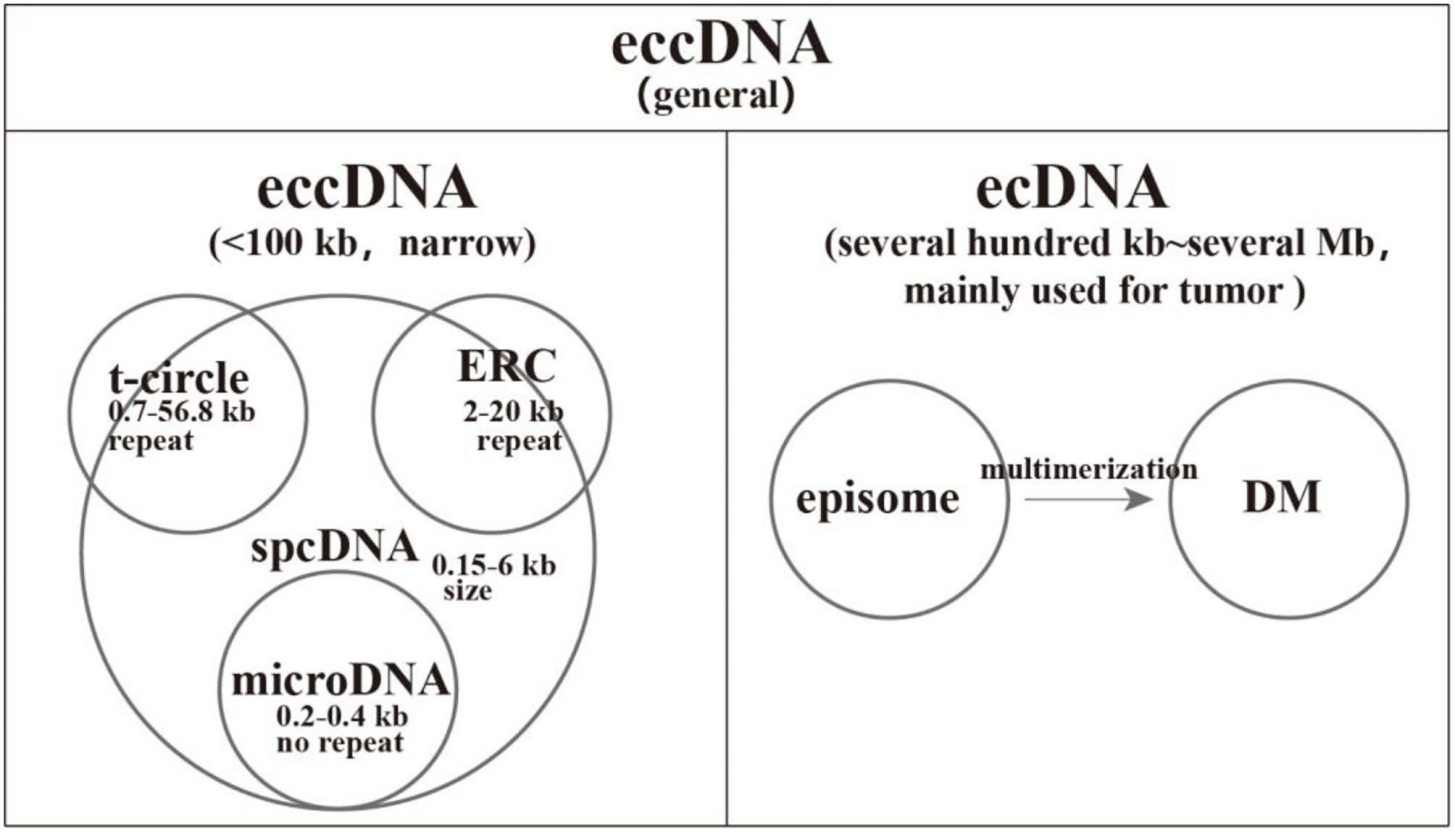
A comprehensive classification of extrachromosomal circular DNAs (eccDNAs). We divide the general eccDNAs into two categories: narrow sense eccDNAs with length < 100 kb and ecDNAs common in tumors with size ranging from several 100 kb to several megabases. Small polydisperse circular DNA (spcDNA) was an obsolete concept to commonly characterize small eccDNAs with repetitive sequences. The length of extrachromosomal rDNA circles (ERCs) and t-circles is larger than that of spcDNA, which means that some ERCs and t-circles were not covered by spcDNA. The deletion of large genomic fragments could be circularized into episomes and subsequently polymerized into double-minutes.

Small polydisperse circular DNA (spcDNA) was an obsolete concept to commonly characterize small eccDNAs with a length of ~0.05–2.00 μm (10). From the 1980s to the 1990s, the repetitive sequences of spcDNAs were widely observed, including short interspersed nuclear element (SINE), long interspersed nuclear element (LINE), tandem repeats, transposons, rDNA, and telomere DNA (11–18). Subsequently, spcDNAs with rDNA and telomere DNA were exclusively termed as extrachromosomal rDNA circles (ERCs) and extrachromosomal telomeric circles (t-circles), respectively. However, their lengths are larger than that of spcDNA, meaning that some ERCs and t-circles are not covered by spcDNA (19–21). With the help of high-throughput sequencing, microDNAs were identified. They have non-repetitive sequences with a length of about 200–400 bp and derive from 5′ UTRs, exons, and CpG islands (22). It is abundant, with several 100 to several 1,000 counts per cell (22, 23). Recently, the concept of extrachromosomal DNA (ecDNA) was developed to exclusively specify larger eccDNA in tumors, typically covering intact oncogenes, and 30% of ecDNAs exist as DMs (24). Further studies revealed that the deletion of large genomic fragments could be circularized into episomes and subsequently polymerized into DMs. Therefore, episomes are the precursors of DMs (25–30). In this review, we divide general eccDNA into two categories: narrow sense eccDNA with length < 100 kb and ecDNA covering DMs and episomes (1, 31, 32). Notably, eccDNA refers to general eccDNA in the following parts unless stated otherwise. Accordingly, we make a clear classification for eccDNAs in Figure 1.

## Biogenesis of EccDNA

**Figure 2:**
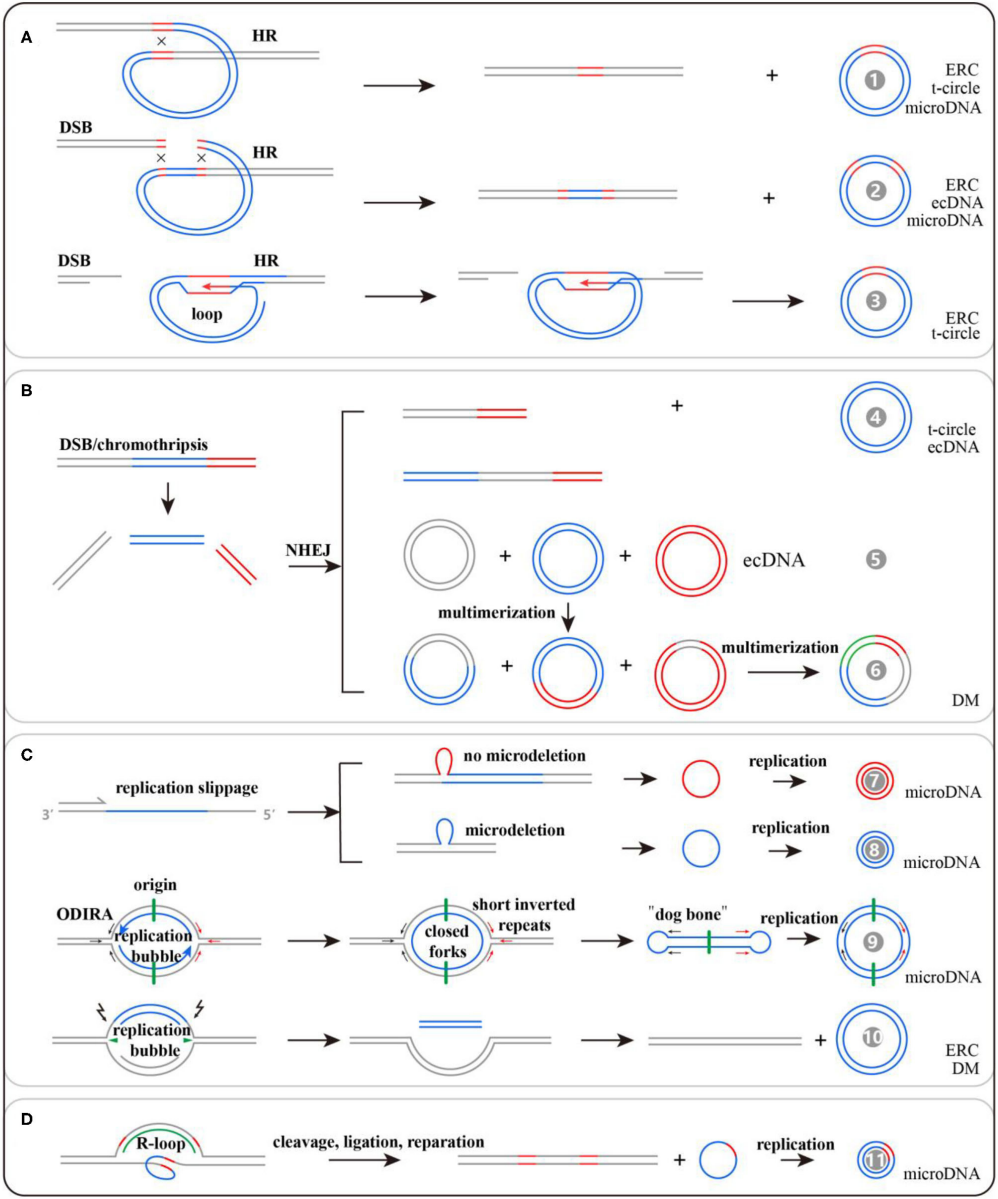
Potential models for extrachromosomal circular DNA (eccDNA) generation: **(A)** formed by HR, **(B)** formed by NHEJ, **(C)** formed by DNA replication, and **(D)** formed by transcription. There are 11 kinds of potential mechanisms for eccDNA formation, and their detailed information can be found in the references listed in Table 1.

Given the heterogeneity of eccDNAs in terms of sequence features, various molecular mechanisms may contribute to eccDNA biogenesis. Interestingly, all these mechanisms seem to be associated with DNA repair (33). We generalize these mechanisms into four categories: homologous recombination (HR), non-homologous end joining (NHEJ), DNA replication, and transcription (Figure 2). However, these potential models underlying the formation of different kinds of eccDNAs require further tests and verification.

DNA double-strand breaks could induce rDNA and tDNA to generate ERCs and t-circles, respectively, *via* loop structures mediated by HR (34). Among the sequencing reads mapped to ~100,000 eccDNAs in human muscle, only 8.1% are from genomic rDNA, 3.5% from LINE, 3.1% from SINE, 1.2% from satellite sequences, and 0.8% from tDNA. Most of the remaining reads are mapped to non-repetitive regions that could produce microDNA (23). The generation of microDNA is strongly associated with DNA mismatch repair. To comprehensively probe microDNA biogenesis, researchers knocked out the key proteins of NHEJ, HR, and mismatch repair (MMR) in a chicken DT40 cell line. They found that knocking out the MMR key protein, MSH3, could cause an 81% decrease of microDNA amount (35). Notably, the microdeletions by MMR were rare, occurring in one of ~400–4,000 alleles from the brain, and would be missed if genomic sequencing was not done at high depth. Nevertheless, the presence of microDNA from >100,000 sites in mouse, human, and chicken cells makes it unlikely that all of them are created by an excision event that leaves behind >100,000 somatically mosaic deletions on the chromosomes (22). Besides this, transcription in GC-rich regions and UTRs could generate triple-stranded DNA/RNA hybrids that function in DNA damage and repair processes and lead to microDNA formation (36). Origin-dependent inverted-repeat amplification may be involved in microDNA production as well. The nascent DNA strand could be circularized *via* short reverted repeats at both ends of the replication bubble (37). Inhibiting the expressions of BRCA1 and PRKDC, the key proteins for HR and NHEJ, respectively, lead to the reduction of ecDNA count in colon cancer cells, suggesting that HR and NHEJ activated by double-strand breaks and macrodeletions may be involved in ecDNA production (38, 39). The summarized mechanisms are listed in Table 1, and detailed information can be obtained from the corresponding references.

**Table 1:** Potential mechanisms of eccDNA formation and corresponding references.

**Number of mechanism**	**EccDNA type**	**Reference type**	**References**	**DOI**
1	ERC	Review	Nelson et al. (40)	https://doi.org/10.1016/j.tig.2019.07.006
		Article	Yerlici et al. (41)	https://doi.org/10.1093/nar/gkz725
	t-circle	Review	Tomaska et al. (42)	https://doi.org/10.1016/j.febslet.2004.04.058
		Article	Yerlici et al. (41)	https://doi.org/10.1093/nar/gkz725
	MicroDNA	Article	Dillon et al. (35)	https://doi.org/10.1016/j.celrep.2015.05.020
		Article	Yerlici et al. (41)	https://doi.org/10.1093/nar/gkz725
2	ERC	Article	Park et al. (43)	https://doi.org/10.1128/MCB.19.5.3848
	ecDNA	Article	Gresham et al. (44)	https://doi.org/10.1073/pnas.1014023107
	MicroDNA	Review	Paulsen et al. (45)	https://doi.org/10.1016/j.tig.2017.12.010
3	ERC	Article	Hull et al. (34)	https://doi.org/10.1371/journal.pbio.3000471
	t-circle	Review	Tomaska et al. (42)	https://doi.org/10.1016/j.febslet.2004.04.058
		Article	Hull et al. (34)	https://doi.org/10.1371/journal.pbio.3000471
4	t-circle	Review	Tomaska et al. (42)	https://doi.org/10.1016/j.febslet.2004.04.058
	ecDNA	Review	Gu et al. (46)	https://doi.org/10.1186/s13046-020-01726-4
		Review	Yan et al. (47)	https://doi.org/10.1186/s13045-020-00960-9
5	ecDNA	Review	Gu et al. (46)	https://doi.org/10.1186/s13046-020-01726-4
		Review	Yan et al. (47)	https://doi.org/10.1186/s13045-020-00960-9
		Review	Liao et al. (48)	https://doi.org/10.1016/j.bbcan.2020.188392
6	DM	Review	Gu et al. (46)	https://doi.org/10.1186/s13046-020-01726-4
		Review	Yan et al. (47)	https://doi.org/10.1186/s13045-020-00960-9
		Review	Liao et al. (48)	https://doi.org/10.1016/j.bbcan.2020.188392
7	MicroDNA	Article	Dillon et al. (35)	https://doi.org/10.1016/j.celrep.2015.05.020
		Review	Paulsen et al. (45)	https://doi.org/10.1016/j.tig.2017.12.010
8	MicroDNA	Article	Dillon et al. (35)	https://doi.org/10.1016/j.celrep.2015.05.020
		Review	Paulsen et al. (45)	https://doi.org/10.1016/j.tig.2017.12.010
9	MicroDNA	Article	Dillon et al. (35)	https://doi.org/10.1016/j.celrep.2015.05.020
		Review	Paulsen et al. (45)	https://doi.org/10.1016/j.tig.2017.12.010
10	ERC	Article	Mansisidor et al. (49)	https://doi.org/10.1016/j.molcel.2018.08.036
	DM	Article	Vogt et al. (50)	https://doi.org/10.1073/pnas.0402979101
		Review	Wei et al. (51)	https://www.ncbi.nlm.nih.gov/pubmed/33294253
11	MicroDNA	Review	Paulsen et al. (45)	https://doi.org/10.1016/j.tig.2017.12.010
		Review	Ain et al. (52)	https://doi.org/10.3390/ijms21072477

## Toolbox For EccDNA Identification

**Figure 3:**
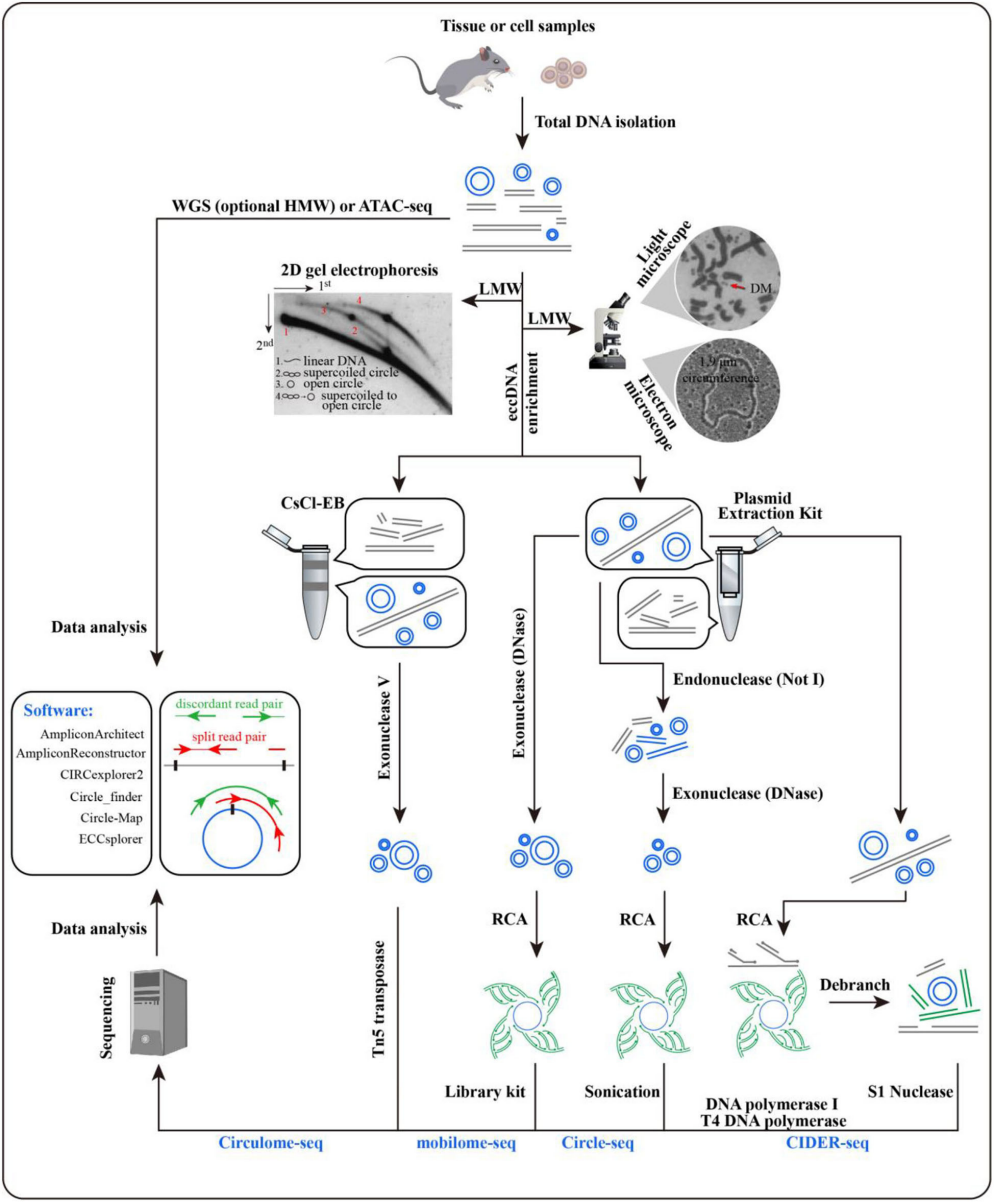
Methods for extrachromosomal circular DNA (eccDNA) identification. Microscopy and electrophoresis are used for eccDNA detection in total DNA after the enrichment of low-molecular-weight DNA [pictures were from Radloff et al. (8), Hahn (53), and Cohen et al. (54)]. Before rolling circle amplification or Tn5 treatment, eccDNAs with target size are enriched by CsCl-EB or a plasmid extraction kit. Split and discordant read pairs are crucial for eccDNA detection, which makes WGS and ATAC-seq data available as well. Various software packages have been developed to call eccDNA with sequencing data, including AmpliconArchitect, AmpliconReconstructor, CIRCexplorer2, Circle_finder, Circle-Map, and ECCsplorer (32, 55–59). Comparisons have been made for some of these packages, and detailed information can be found in Prada-Luengo et al. (57).

Large eccDNAs could be observed with a light microscope in karyotype analysis, but as for the small eccDNAs, electron microscopy is necessary, and their lengths can be estimated all at once (Figure 3) (8, 53). This estimation could be achieved by 2D electrophoresis as well, but its detection power ranges from 0.7 to 56.8 kb (19). Southern blotting enables 2D electrophoresis to reveal the sequence features of eccDNA (60). Interestingly, software, such as ECdetect, has been developed for moderate-through counting of eccDNAs in DAPI-stained cells (24).

Initiated with a large amount of cells, the CsCl-EB method is time consuming and labor intensive for eccDNA enrichment, where most nicked circles are missed. Therefore, this approach is being replaced by plasmid extraction kits (60–62). The enriched eccDNAs can be subjected to high-throughput sequencing to determine their locations and junction sites by Circulome-seq, mobilome-seq, Circle-seq, or CIDER-seq (Figure 3). Circulome-seq adopts Tn5 transposition-based fragmentation and a tagging system, which simplify the sequencing library construction. This method could detect eccDNAs in length ranging from several hundreds of base pairs to several hundreds of kilobase pairs (62). Mobilome-seq is highly suitable for the detection of retrotransposon eccDNAs. It removes linear DNA with exonuclease DNase and then performs rolling circle amplification (RCA) (63). Circle-seq combines endonuclease *Not*I with exonuclease DNase to fully eliminate linear DNA, potentially leading to an unexpected damage on eccDNAs; its detection power is ~1–38 kb (64). As for CIDER-seq, RCA is performed straightforwardly for the eccDNAs enriched by plasmid extraction kits without the removal of linear DNA. Given the undesired amplification of linear DNA, long-read single-molecule real-time sequencing is used to guarantee the amount of valid reads (i.e., split and discordant reads), which are essential for eccDNA calling (65). CIDER-seq has a similar power to Circulome-seq, but the former has a more accurate detection for <10 kb eccDNAs. Notably, it is advisable to introduce plasmids as a control or internal reference during eccDNA library construction.

Despite the above-mentioned methods, whole-genome sequencing (WGS) and assay for transposase-accessible chromatin using sequencing (ATAC-seq) can be used for eccDNA calling, given the presence of split and discordant reads in their sequencing data (24, 66, 67). It has been demonstrated that 100% of ecDNAs and 30% of eccDNAs (narrow sense) called from WGS were supported by Circle-seq (32). Prior enrichment of high-molecular-weight DNA is helpful in ecDNA identification (1). To date, various software packages have been developed to call eccDNA with sequencing data (Figure 3).

### Advances of EccDNA Function

EccDNA are able to self-replicate (unknown for microDNA) and evenly segregate to daughter and germ cells during mitosis and meiosis, respectively, because of lacking centromeres. Some eccDNAs can be reintegrated into genomic homogeneously staining regions (HSRs). The strong accessibility of ecDNA leads to highly frequent interactions between regulatory elements. These features equip cells with high heterogeneity and environmental adaptability (1, 31, 68).

## Telomere and rDNA Copy Number Maintenance

Telomeric arrays can be maintained through various mechanisms, such as telomerase activity or recombination. T-circles function in recombination-dependent maintenance pathways by serving as templates for the rolling circle synthesis of telomere DNA. This may be the same case for animal and plant t-circles (20, 69). It is estimated that 15% of human immortalized cell lines may maintain telomere length through alternative lengthening of telomeres (70). A total of ~100–1,000 copies of eukaryotic rDNA are tandemly organized in the genome to satisfy the need for ribosome synthesis. The production of ERC reduces the copy number of rDNA in the *Drosophila* genome that could recover in germ cells. Studies have found that ERC could reintegrate into the genome to maintain rDNA copy number by self-replication (40).

## Aging

Asymmetric inheritance and self-replication lead to ERC accumulation in yeast mother cells. The number of ERCs per mother cell after 15 generations has been estimated at between 500 and 1,000. Mutations in *SGS1* could result in ERC accumulation and age-associated phenotypes in addition to a shortened lifespan. Conversely, loss of the replication fork blocking protein Fob1 decreases the formation of ERCs and extends the lifespan of mother cells by 30–40% (71). Interestingly, genes present on low-copy plasmids confer fitness effects rarely and of much lower magnitude than those on multi-copy plasmids. Therefore, young yeast populations contain about 1,800 circular DNA species, and it is only with substantial accumulation of any given circular DNA that major phenotypic effects are likely to manifest (64, 71, 72). These facts demonstrate that ERC accumulation functions in yeast aging. It was proposed that yeast senescence may be the result of health sacrifice to environmental adaption by accumulating specific eccDNAs, which could titrate components of the replication or transcription machinery and lead to an inability to replicate or transcribe genomic DNA and, thus, growth arrest and eventual death (5). According to this assumption, *CUP1* eccDNA enrichment in a CuSO_4_ environment may contribute to yeast aging, but further research is still necessary (34).

## Tumorigenesis and Drug Resistance

DMs, typically bearing intact oncogenes, are tumor specific and have been detected in 182/200 kinds of tumors, and ~0.26–44% of cancer patients and 7–100% of cancer cells have DMs (50, 53, 73–79). ecDNA exclusively refers to larger eccDNA in tumors where 30% of ecDNAs existed as DMs (24). The survival rate of cancer patients with ecDNAs is significantly lower than those without ecDNAs, making ecDNA a potential prognostic marker (80, 81). As a carrier of oncogene amplification, ecDNAs are subjected to non-Mendelian inheritance, which enables tumors to achieve very high intratumoral genetic heterogeneity and evolve rapidly in response to changing conditions (24, 66, 82)—for example, *EGRF, MET*, or *MYC* ecDNAs can make tumor cells proliferate rapidly and further develop into tumor invasion and migration (51, 66). There is a substantial quantity of cell-free microDNAs in the plasma and serum from both healthy individuals and cancer patients (3, 4). Surprisingly, tumor-derived human microDNAs are detected in mouse circulation in a mouse xenograft model of human ovarian cancer, and tumor excision alters the length of these small molecules (3). Thus, circular DNA in the circulation is a previously unexplored pool of nucleic acids that could complement miRNA and linear DNA for diagnosis and intercellular communication (3).

It had been accepted that the high copy number of ecDNA leads to oncogene overexpression. However, the significant transcriptional activity of ecDNA does matter as well (1). The deficiency of chromatin high-order structure and suppressing histone modification make ecDNAs more accessible than their genome parallels, which instigates strong promoter–enhancer interactions. Furthermore, ecDNA enhancers can shake off the insulator shackles and lead to novel interactions with oncogenes, which causes additional expression (83, 84). More recently, researchers have reported the chromatin connectivity networks of ecDNA in cancer, revealing that ecDNA can function as mobile super-enhancers, which drive genome-wide transcriptional amplification, including that of oncogenes. These findings support an expanded role for ecDNA in trans-regulating chromosomal genes in promoting tumor growth (2).

*EGFR* VIII, an oncogenic variant, could accelerate glioblastoma growth, but it also makes cells more sensitive to the EGFR tyrosine kinase inhibitor (TKI) (85). After TKI treatment, the proportion of TKI-sensitive tumor cells with a high expression of *EGFR* VIII was significantly decreased, whereas cells with low *EGFR* VIII expression were increased (85). Studies have demonstrated that tumor TKI resistance is caused by the elimination of DMs containing *EGFR* VIII, which could reintegrate into the genome HSRs (86). However, after drug withdrawal, the reemergence of clonal *EGFR* mutations on ecDNA follows quickly (86). Through this mechanism, cancer cells can escape targeted oncogene therapy. Therefore, pulsatile intermittent treatment with much higher doses of TKI could potentially lead to better target inhibition and even possibly less toxicity relative to continuous dosing (24). Notably, the self-replication of *EGFR* DMs could also generate *EGFR* mutations, which would provide additional heterogeneity (86).

## Phenotypic Effects on Animals and Plants

To our current knowledge, eccDNAs have been associated with animal phenotypes, including cattle color sideness (Cs) and pigeon muscle development. Cs is a dominantly inherited trait characterized by a white band along cattle spines. The dominance of the *Cs* allele is expected to reflect a gain of function resulting from the dysregulated expression of the translocated *KIT* gene. A 492-kb fragment containing the *KIT* gene on chromosome 6 produces a circular intermediate (now referred to as eccDNA) and integrates into chromosome 29 to form the *Cs29* allele. Then, a 575-kb fragment containing the partial *Cs29* allele is circularized and translocated to chromosome 6 as *Cs6* allele (87). Regenberg et al. found that the number of eccDNA in king pigeon muscle is nine-fold higher than that of homing pigeons. Interestingly, eccDNAs bearing the *AGRIN* gene were identified (88). This gene encodes a membrane protein that is involved in the development of neuromuscular junctions, and its variations could lead to abnormal muscle development (88).

As for plants, *Amaranthus palmeri* can develop herbicide resistance to glyphosate by amplification of the *EPSPS* gene as eccDNA, the molecular target of glyphosate. These circular molecules can be transmitted to germ cells and drive rapid glyphosate resistance through genome plasticity and adaptive evolution (89, 90). Moreover, retrotransposons, such as *EVD* and *Tos17*, can produce eccDNAs and insert into the genome to improve the response to environmental stress through promoting DNA methylation and gene silencing at the transcriptional level (63).

**Figure 4:**
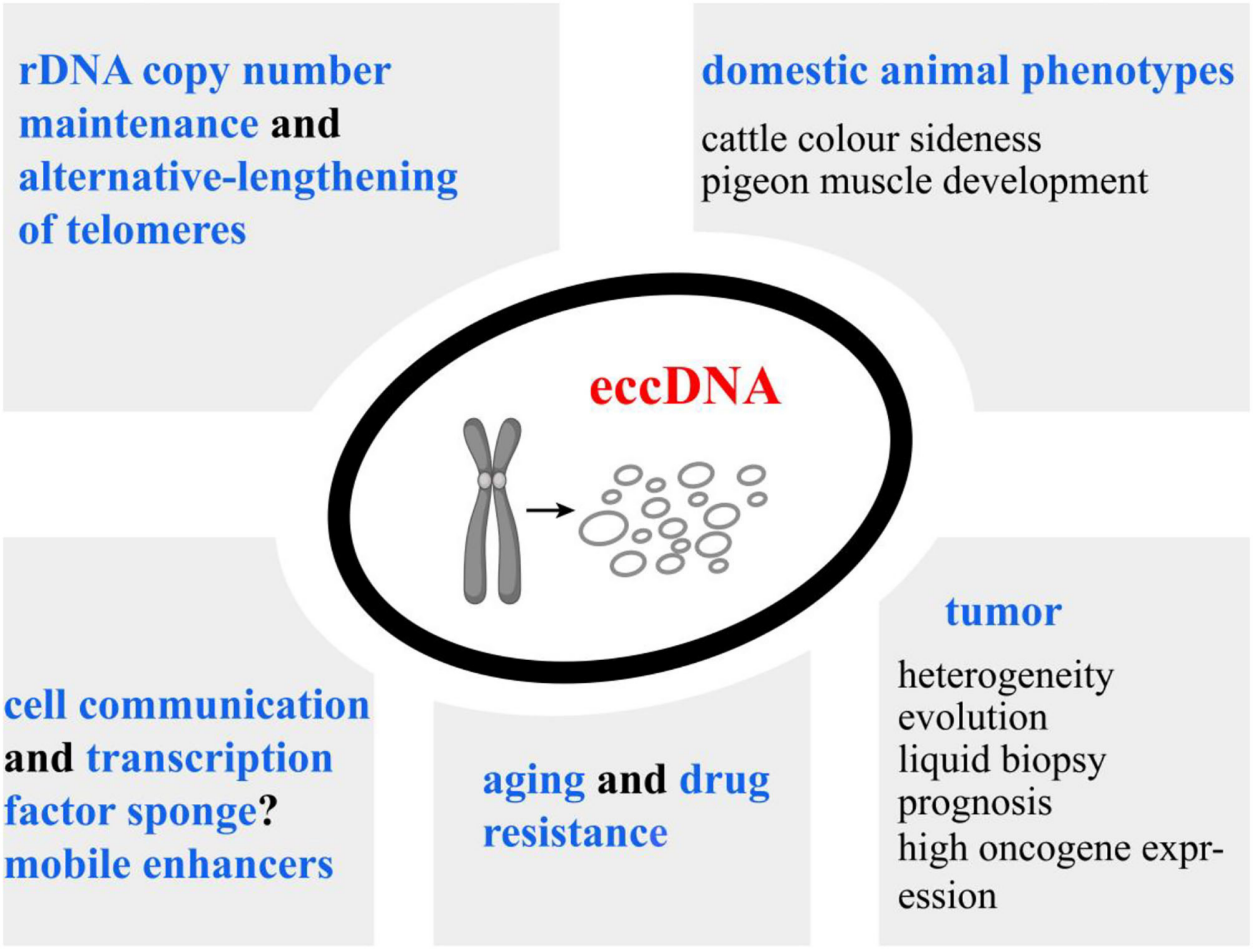
Overview of our current understanding of extrachromosomal circular DNA (eccDNA) functions. High copy number and significant transcriptional activity of eccDNAs lead to the overexpression of the inhabiting genes. Additionally, eccDNAs could serve as mobile enhancers to trans-regulate chromosomal genes. eccDNAs have been associated with cancer prognosis, drug resistance of plants, and phenotypic variations of animals, implying their potential implications in life science. MicroDNA can be released from normal tissues. Establishing the associations of microDNA copy numbers with economic traits is an important direction for future exploration.

The above-mentioned facts suggest that eccDNAs may be promising molecular markers in life science. However, the inability to provide biopsies of some tissues limits the use of large eccDNAs as biomarkers. MicroDNAs released to the circulation represent a previously unexplored pool of nucleic acids; although they are too small to contain protein-coding genes, they are sufficiently long to code for regulatory elements. Possible acting mechanisms of microDNA have been assumed based on indirect evidence, including cell communication, transcription factor sponges, and mobile enhancers to trans-regulate chromosomal genes (Figure 4). Thus, establishing the associations of microDNA with a particular phenotype or disease is an important direction for future exploration.

## Conclusions and Perspectives

The occurrence of eccDNA is a ubiquitous, normal phenomenon in eukaryotic systems, including those of plants, yeasts, and animals. There are various types of eccDNA according to sequence feature and size. Several models have been proposed to explain the formation and proliferation of eccDNAs, but the underlying mechanisms and direct evidence for each model are still required. Regardless of the above-mentioned issues, the CRISPR-hapC system has been developed for genome haplotyping based on the generation of eccDNA in cells (91). This system can map haplotypes from a few 100 bases to over 200 Mb and will be important for genome research and haplotype-specific gene therapy.

ecDNA could drive oncogene amplification and has become a hotspot of research in tumor pathogenesis and evolution. Additionally, the survival rate of cancer patients with ecDNAs is significantly lower than those without ecDNAs, making ecDNA a potential prognostic marker (80, 81). However, an interesting question arises: Are tumor suppressor genes present in ecDNA? If they exist, what are their functions? In plants and animals, large eccDNAs bearing intact genes have been identified, and they play important roles in environmental stress response and phenotypic variations, respectively (87, 89, 90). These facts make eccDNA a particularly promising molecular marker for breeding. However, given the unavailability of some tissue biopsies, it may be difficult to use large eccDNAs as biomarkers for early diagnosis and breeding.

MicroDNA represents the majority of eccDNAs. It has been detected as abundant cell-free DNA in plasma and serum released both by normal and tumor tissues (3, 4). Thus, establishing the associations between microDNAs and economic phenotype or diseases is an important direction for future exploration. Fortunately, an eccDNA database (eccDNAdb, http://www.eccdnadb.net/) has been set up. It has recorded a total of 1,700,000 eccDNAs for humans, mice, and chickens, which will provide supporting data for association analyses. If there are positive results, then the mechanisms of their regulatory roles can be validated, including serving as sponges of transcription factors, carriers of regulatory RNAs in intercellular communication, or mobile enhancers to globally amplify chromosomal transcription. These mechanisms may also provide novel insights into the phenotypic effects of genome copy number variations (41, 45, 92, 93). All of these questions require a further in-depth exploration.

## Author Contributions

XC proposed the topic, retrieved literatures, provided outline, tables, and figures, and revised the manuscript. SW, LG, and WZ wrote the manuscript. JH and WS reviewed the final manuscript. All the authors read and approved the final manuscript.

## Funding

This work was supported by the Key Research and Development Plan (modern agriculture) in Jiangsu Province (BE2018354), Jiangsu Agricultural Science and Technology Innovation Fund [XC(18)2003], major projects of Natural Science Research of colleges and universities in Jiangsu Province (17KJA230001), National Natural Science Foundation of China (31872333), major new varieties of agricultural projects in Jiangsu Province (PZCZ201739), National Natural Science Foundation of Cooperation on International Agricultural Research Organization (32061143036), and Open Project of Joint International Research Laboratory of Agriculture and Agri-Product Safety and the Ministry of Education of China (JILAR-KF202103).

## Conflict of Interest

The authors declare that the research was conducted in the absence of any commercial or financial relationships that could be construed as a potential conflict of interest.

## Publisher's Note

All claims expressed in this article are solely those of the authors and do not necessarily represent those of their affiliated organizations, or those of the publisher, the editors and the reviewers. Any product that may be evaluated in this article, or claim that may be made by its manufacturer, is not guaranteed or endorsed by the publisher.

## Abbreviations

ALT: alternative lengthening of telomeres
ATAC-seq: transposase-accessible chromatin using sequencing
BRCA1: BRCA1 DNA repair-associated
CsCl-EB: cesium chloride ethidium bromide
CUP1: copper resistance-associated metallothionein
DAPI: 4′,6-diamidino-2-phenylindole
DM: double-minute
EccDNA: Extrachromosomal circular DNA
ecDNA: extrachromosomal DNA
EGRF: epidermal growth factor receptor
EPSPS: 3-phosphoshikimate 1-carboxyvinyltransferase 2
ERC: extrachromosomal rDNA circle
HMW: high molecular weight
HR: homologous recombination
HSR: homogeneously staining region
KIT: KIT proto-oncogene, receptor tyrosine kinase
LINE: long interspersed nuclear element
LMW: low molecular weight
MET: MET proto-oncogene, receptor tyrosine kinase
MMR: mismatch repair
MSH3: MutS Homolog 3
MYC: MYC proto-oncogene, bHLH transcription factor
NHEJ: non-homologous end joining
ODIRA: origin-dependent inverted-repeat amplification
PRKDC: protein kinase, DNA-activated, catalytic subunit
RCA: rolling circle amplification
rDNA: ribosomal DNA
SGS1: ATP-dependent DNA helicase SGS1
SINE: short interspersed nuclear element
SMRT: long-read single-molecule real-time sequencing
spcDNA: small polydisperse circular DNA
t-circle: extrachromosomal telomeric circle
tDNA: telomere DNA
TKI: EGFR tyrosine kinase inhibitor
WGS: whole genome sequencing.
