# Corrigendum: Extrachromosomal Circular DNA: Category, Biogenesis, Recognition, and Functions

**DOI:** 10.3389/fvets.2021.784611

**Published:** 2021-10-27

**Authors:** Xiukai Cao, Shan Wang, Ling Ge, Weibo Zhang, Jinlin Huang, Wei Sun

**Affiliations:** ^1^Joint International Research Laboratory of Agriculture and Agri-Product Safety of Ministry of Education of China, Yangzhou University, Yangzhou, China; ^2^College of Animal Science and Technology, Yangzhou University, Yangzhou, China; ^3^Jiangsu Key Laboratory of Zoonosis, Yangzhou University, Yangzhou, China

**Keywords:** eccDNA, microDNA, tumor, livestock, molecular marker

There is an error in the Funding statement. The correct number for National Natural Science Foundation of Cooperation on International Agricultural Research Organization is 32061143036. The corrected Funding statement appears below.

## Funding

This work was supported by the Key Research and Development Plan (modern agriculture) in Jiangsu Province (BE2018354), Jiangsu Agricultural Science and Technology Innovation Fund [XC(18)2003], major projects of Natural Science Research of colleges and universities in Jiangsu Province (17KJA230001), National Natural Science Foundation of China (31872333), major new varieties of agricultural projects in Jiangsu Province (PZCZ201739), National Natural Science Foundation of Cooperation on International Agricultural Research Organization (32061143036), and Open Project of Joint International Research Laboratory of Agriculture and Agri-Product Safety and the Ministry of Education of China (JILAR-KF202103).

The authors apologize for this error and state that this does not change the scientific conclusions of the article in any way. The original article has been updated.

## Publisher's Note

All claims expressed in this article are solely those of the authors and do not necessarily represent those of their affiliated organizations, or those of the publisher, the editors and the reviewers. Any product that may be evaluated in this article, or claim that may be made by its manufacturer, is not guaranteed or endorsed by the publisher.

